# Boylston Prize Dissertations for the Years 1836 and 1837

**Published:** 1838-06-06

**Authors:** 


### BIBLIOGRAPHICAL NOTICE.

*Boylston Prize Dissertations for the years* 1836 *and* 1837. By Oliver Wendell Holmes, M. D., Fellow of the Massachusetts Medical Society, and Member of the Societé Médicale d'Observation of Paris. Boston: Charles C. Little and James Brown. MDCCCXXXVIII. 8vo. pp. 371.

The perusal of this exceedingly neat volume has afforded us much pleasure; not only from its intrinsic merit, which is of no mean order, but as the evidence of talent and industry, from which we may expect further valuable contributions to our national medical literature. Dr. Holmes is one of those young American physicians who completed their initiatory studies in Paris, under the immediate eye of the founder of the Numerical Method, and his first literary effort is abundant proof that he has profited amply by the example and instruction of his distinguished preceptor.

The three essays, which form the volume before us, obtained the Boylston Prize for two successive years, and are calculated to impress very favourably the reader with the diligence, research, and abilities of the author, and entitle him to be considered, in every respect, as a very deserving disciple of the school, under whose auspices he was educated.

The first dissertation is styled "*Facts and Traditions respecting the existence of Indigenous Intermittent Fever in New England.*" In this essay there is compressed in a small compass a vast amount of information, evidently the result of much labor and investigation, much of which must have been uninteresting and profitless. In entering upon this subject, Dr. Holmes had an untrod country to explore, with no landmarks from previous voyagers to cheer him on his way, or assist him in determining his positions. Arid and dubious as was his path, he has contrived to collect much that is curious and valuable, respecting the local medical history of New England, and, moreover, from his unambitious and sensible mode of treating what many might be disposed to deem wearisome and sectional, has produced by no means an uninteresting treatise.

The second dissertation is upon "Neuralgia," and evinces an intimate acquaintance with the bibliography of that protean malady, as well as considerable judgment in the collation. Much originality could not be expected, nor is any pretension made to it. Dr. Holmes has given us what was very much wanted, a concise history of this common affection, with an unbiassed and succinct account of the views entertained by the different authors, as to the nature, treatment, &c. of the disease.

The third, and concluding dissertation is, "On the utility and importance of direct Exploration in Medical Practice," and, to our mind, the most valuable in the collection. The importance of direct exploration of the organs in the treatment of disease is now so universally, and so justly, conceded, that it is difficult to reconcile with this due appreciation the neglect it meets with among practitioners, immediately removed from the head-quarters of medicine. Yet such, unfortunately, is the case. Every physician, in this city, is often meeting with cases, from the surrounding districts, which, had not the general symptoms been relied on to determine the diagnosis, might never, probably, have progressed to the formidable and often intractable condition in which they find them. We feel that we cannot impress with too much earnestness, upon the consideration of our distant readers, this point, and we rejoice in the publication of every work which treats upon the subject, and is calculated to make it more popular and useful.

